# Effect of thyroid function on assisted reproduction outcomes in euthyroid infertile women: A single center retrospective data analysis and a systematic review and meta-analysis

**DOI:** 10.3389/fendo.2022.1023635

**Published:** 2022-10-10

**Authors:** Viviane Rosado Negreiros d’Assunção, Erik Montagna, Luis Eduardo Negreiros d’Assunção, Maria Madalena Pessoa Caldas, Denise Maria Christofolini, Caio Parente Barbosa, Ricardo Andre Medeiros Negreiros, Antonio Simone Laganà, Renato de Oliveira, Bianca Bianco

**Affiliations:** ^1^ Postgraduation Program in Health Sciences, Faculdade de Medicina do ABC, Santo André, Brazil; ^2^ Centre for Medical Sciences, Federal University of Paraíba, Paraíba, Brazil; ^3^ Geare Centro de Reprodução Humana e Genética, Pernambuco, Brazil; ^4^ Discipline of Sexual and Reproductive Health, and Populational Genetics, Department of Collective Health, Faculdade de Medicina do ABC, Santo André, Brazil; ^5^ Unit of Gynecologic Oncology, Azienda di Rilievo Nazionale ed Alta Specializzazione Ospedali Civico Di Cristina Benfratelli (ARNAS) “Civico – Di Cristina – Benfratelli”, Department of Health Promotion, Mother and Child Care, Internal Medicine and Medical Specialties (PROMISE), University of Palermo, Palermo, Italy; ^6^ Department of Urology, Instituto Israelita de Ensino e Pesquisa Albert Einstein, São Paulo, Brazil

**Keywords:** assisted reproductive technology, infertility, *in vitro* fertilization, pregnancy rates, thyroid, TSH

## Abstract

**Background:**

The influence of thyroid-stimulating hormone (TSH) on gestational outcomes have been studied and checked whether differing TSH levels are relevant on human reproduction outcomes. International guidelines recommend TSH values <2.5 mIU/L in women trying to conceive, since values above this level are related to a higher frequency of adverse reproductive outcomes. This study aimed to evaluate whether TSH values correlate with different gestational outcomes in euthyroid infertile women without autoimmune thyroid disease.

**Methods:**

A retrospective cohort study was conducted involving 256 women who underwent *in vitro* fertilization (IVF) treatment. The participants were divided into two groups: TSH 0.5-2.49 mIU/L (n=211) and TSH 2.5-4.5 mIU/L (n=45). The clinical data, hormonal profiles and reproductive outcomes were compared between groups. Additionally, a systematic review with meta-analysis following the PRISMA protocol was carried out in PubMed/MEDLINE, EMBASE, and SciELO, with no time or language restrictions, for articles comparing TSH groups named “low TSH” (<2,5 mIU/L) and “high TSH” (≥2.5 mIU/L). A meta-analysis of proportions was performed with pooled estimates expressed as relative risk (RR) of events and a random effects model.

**Results:**

Age, BMI, free thyroxine levels (FT4) hormonal profile and IVF outcomes were not different between groups, neither gestational outcomes (p=0.982). Also, no difference was observed when the TSH and FT4 levels were compared between patients with positive or negative gestational outcomes (p=0.27 and p=0.376). Regarding the systematic review with meta-analysis, 17 studies from 2006 to 2022 were included, and added by this original retrospective research comprising 13.247 women undergoing IVF. When comparing the proportions of clinical pregnancy between the TSH groups, no significant difference was found (RR 0.93, 95% CI 0.80–1.08), with high between studies heterogeneity (*I²*: 87%; τ^2^: 0.0544; p<0.01). The number of deliveries was not significantly different between groups, despite a trend towards higher frequency in the high-TSH group (RR 0.96, 95% CI 0.90–1.02).

**Conclusion:**

Variation in TSH levels within the normal range was not associated with pregnancy and delivery rates in women, without autoimmune thyroid disease, who underwent IVF treatment.

**Systematic review registration:**

https://www.crd.york.ac.uk/prospero/, identifier CRD 42022306967.

## Introduction

A healthy function of the maternal thyroid is extremely important during the early gestational development, because the child’s thyroid gland only fully develops between the 18^th^ and the 20^th^ week of gestation. The mother is responsible for supplying the needs of the embryo and the foetus for thyroid hormones until the gland matures (1, 2). Maternal thyroid hormones such as free thyroxine (FT4) are also involved in the mechanisms of metabolic exchange in pre-implantation embryos (3). Thyroid-stimulating hormone (TSH) may also influence reproductive outcomes directly, since TSH receptors are present in granulosa cells and in the ovarian stroma, as well as in the human endometrium, especially during folliculogenesis (1).

TSH at serum concentrations above reference values (0.5–4.5 mIU/L) has been associated with adverse reproductive and pregnancy outcomes, including infertility, miscarriages, prematurity, and congenital defects, even when FT4 is within values (2, 4, 5). International guidelines on recurrent miscarriage uniformly recommend that TSH levels should be <2.5 mIU/L in women with this diagnosis and hypothyroidism (6). Nevertheless, the effect of TSH variations within the reference values on reproductive outcomes is still unclear in patients without the aforementioned diagnoses.

TSH and FT4 are used as the main parameters to evaluate thyroid function, taking into account that both undergo changes during pregnancy, and therefore their concentrations must have been well established before the gestational period (7, 8). Accumulating evidence suggests that for patients with subclinical hypothyroidism undergoing assisted reproductive techniques, the ideal serum TSH value is <2.5 mIU/L, which can be obtained by treatment with levothyroxine (LT4) if necessary (6, 9). However, the use of LT4 in patients negative for thyroid antibodies and with TSH within the normal range is controversial, even if TSH value >2.5 mIU/L (9).

There are treatment recommendations for TSH values >4 mIU/L; however, for values between 2.5 and 4.0 mIU/L, replacement therapy with LT4 is still considered, despite inconsistent or limited evidence reporting interference with gestational outcomes in this TSH range (9, 10).

In this context, a retrospective cohort study was conducted to evaluate whether the serum TSH values and other variables of interest are related to the gestational outcomes in euthyroid women without autoimmune diseases who underwent *in vitro* fertilisation/intracytoplasmic sperm injection (IVF/ICSI). The present original study was also followed by a systematic review of the literature with meta-analysis to investigate a possible association of TSH values in the 0.5–2.49 mIU/L and 2.5–4.5 mIU/L ranges with gestational outcomes in women undergoing IVF.

## Methods

### Retrospective study

#### Study design

This retrospective study was conducted at the Geare Centre for Reproductive Medicine in Recife, Brazil, with data from patients seen between January 2010 and December 2017. The Research Ethics Committee of the Centre for Medical Sciences of the Federal University of Paraíba (approval code CAAE 25654719.5.0000.8069) was approved before to start the enrolment. Considering the retrospective nature of the study, and the consistency with the REporting of studies Conducted using Observational Routinely-collected health Data (RECORD) Statement without any experimental intervention by the researchers, and the anonymized data collection, a formal informed consent was waived from by the Research Ethics Committee. The study was not advertised, and no remuneration was offered to the patients to enter or continue the study. An independent data safety and monitoring committee evaluated the ad interim and final results of this study. Over the study period, there were no significant differences in the facilities available for patient care and in the referral patterns of our service.

#### Patients and population

All the women evaluated in this study underwent IVF/ICSI. The clinical data, hormonal profiles, and reproductive outcomes relevant to the research were extracted from the participants’ medical records. The inclusion criteria were women aged ≤40 years, follicle-stimulating hormone (FSH) ≤12.0 IU/L, TSH ≥0.5 mIU/L and ≤4.5 mIU/L, both ovaries present without morphological abnormalities, and no history of endocrine diseases, such as hyperprolactinemia and polycystic ovary syndrome. The exclusion criteria were patients with autoimmune disease, with previous history of ovarian surgery, who had undergone radio/chemotherapy, or who had received previous hormonal treatment, including Levothyroxine Sodium (LT4) tablets.

The sample size used was based on convenience, according to the digital medical records database available to the authors. A *post-hoc* power analysis, considering a two-tailed test, with an effect size of 0.5 and alpha 0.05 that resulted in a Power (1 – beta) of 0.843.

The cause of infertility was investigated according to established diagnostic procedures for infertile couples, including semen analysis, serological tests, hormonal profile, thyroid peroxidase antibody test, tests for sexually transmitted infections, and imaging examinations (trans-vaginal ultrasonography, hysterosalpingography, and hysteroscopy) (11, 12). Hysteroscopy is a routine examination at the study clinic, where it is considered a part of the minimal diagnostic procedures for infertility. It was included in the study because it is the gold standard examination for evaluating the uterine cavity.

#### Hormone measurement

The serum FSH and luteinising hormone (LH) concentrations were obtained during the follicular phase of the menstrual cycle, whereas the prolactin levels were measured during the luteal phase. The serum levels of TSH, FT4 were detected before controlled ovarian stimulation (COS) for IVF/ICSI.

#### Study procedure

Controlled ovarian stimulation (COS) was initiated between the first and third day of menstrual cycle using follitropin alpha 300 IU/day + lutropin alpha 150 IU/day (Pergoveris, Merck, Germany) for an average period of 10 days. From the sixth day of COS, when at least one follicle with a minimum diameter of 14 mm was identified, a gonadotropin release hormone (GnRH) antagonist was initiated, namely cetrorelix acetate 0.25 mg/day (Cetrotide, Merck, Germany), concomitantly with the use of gonadotropin. When at least three follicles of size 17–22 mm were identified, oocyte maturation was triggered with GnRH analogues leuprorelin acetate 1 mg subcutaneously (Lupron, Abbott, USA). After 36 h, an ovarian puncture was performed for oocyte retrieval.

Four hours after oocyte retrieved, IVF/ICSI was performed, and the embryos were kept in a culture medium until the blastocyst stage, lasting between 5 and 7 days. The embryos were subsequently frozen by the vitrification method.

The patients were prepared for the embryo transfer with oestradiol valerate 6 mg/day (Primogyna, Bayer, Germany) starting between the first and third day of the menstrual cycle, after ultrasonographic evaluation. After 10–12 days, a new transvaginal ultrasonographic evaluation was performed. Additionally, if the endometrial thickness was above 7 mm, oestradiol levels were between 200 and 300 pg/mL, and the progesterone levels were <1 ng/mL, then vaginal progesterone 800 mg/day (Utrogestran, Besins Health Care, France) was started 5 days prior to the transfer of the thawed embryo. A maximum of two embryos were transferred in each procedure.

The primary gestational outcome was clinical pregnancy, confirmed by the presence of a gestational sac and foetal heartbeat by a transvaginal ultrasound performed four weeks after the embryo transfer. The secondary gestational outcome was the number of live births.

#### Statistical analyses

Statistical analyses were performed using RStudio software, version 1.1.383 (The R Foundation for Statistical Computing, Vienna, Austria). Data normality was assessed using the Shapiro–Wilk test. The descriptive analysis was presented as absolute and relative frequencies, and numerical variables were presented as medians and interquartile ranges (IQR), as none of the data presented normal distribution. Either the Mann–Whitney U test or the chi-square test was used to analyse the differences in the hormone levels and reproductive outcomes between the groups. Logistic regression was used considering the primary and secondary outcomes as response variables. The level of statistical significance was set at p<0.05.

### Systematic review and meta-analysis

A systematic review was conducted according to the Preferred Reporting Items for Systematic Reviews and Meta-Analyses (PRISMA) (13) guidelines. This study is registered in International Prospective Register of Systematic Reviews (PROSPERO; CRD 42022306967). The study question is whether the levels of TSH are determinant of clinical pregnancy as the main outcome of IVF in patients with higher TSH levels (≥2.5 mIU/L) compared to low TSH levels (<2.50 mIU/L).

#### Search strategy and selection criteria

We selected observational studies with women undergoing IVF/ICSI for those were available data about treatment outcomes and hormonal profiling that presented dosages of at least TSH and FSH for subgroups with different TSH levels. The exclusion criteria were studies comprising patients with autoimmune disease or were under hormonal treatment for thyroid diseases. The primary outcome was clinical pregnancy, and the secondary outcome was the number of live births.

The databases searched were MEDLINE, EMBASE, and SciELO using their respective search engines.

The search term and the combination were ((“Thyroid”(Title/Abstract) OR “TSH”)(Title/Abstract) AND (“infertility”(Title/Abstract) OR “IVF”(Title/Abstract) OR “*In Vitro* Fertilization”(Title/Abstract) OR “ICSI”(Title/Abstract) OR “Intracytoplasmic sperm injection”(Title/Abstract) OR “Assisted reproduction techniques”(Title/Abstract) OR “ART”)(Title/Abstract)) NOT (“autoimmunity” OR “cancer” OR “neoplasia” OR “neoplasm” OR “carcinoma”). Search strategy for SciELO is closely related to the MEDLINE. Search strategy for Embase is presented as Supplement.

Titles and/or abstracts of studies retrieved were screened independently by two review authors (BB and EM) to identify studies that potentially meet the aims of the systematic review. The full text was retrieved and independently assessed by two authors (EM and RAMN). Any disagreement over the eligibility articles was resolved through discussion with a third collaborator (CPB). No translation was needed during the process. All the process was performed without any kind of automated or machine learning process.

#### Data extraction

Data extraction was performed using an electronic form by two independent investigators (RN and LE), with disagreements resolved by a third investigator (VR). No automation tools were used.

The data retrieved were author and date, study design, total and stratified sample in the study groups, primary and secondary outcomes of the groups of interest. No data imputing was performed.

#### Data synthesis

Studies considered eligible for synthesis were those containing data according to the eligibility criteria of patients and that were possible to extract data, disregarding the primary study aim. No restriction to language or publication date was imposed, retrieveing studies up to May 18^th^, 2022.

The absolute number of observed events was used to calculate the proportions and the random effects model. The measures of concentration were standardised to the same unit, and the values were transformed into means and standard deviations when presented in a different unit (14).

The data was presented in a summary of evidence and synthesis as forest plots, with studies ordered by publication year.

Two analyses were performed. The first one compared groups according to the TSH level, <2.50 mIU/L (labeled ‘low TSH’) and ≥2.5 mIU/L (labeled ‘high TSH’) as thresholds, considering clinical pregnancy as the main outcome, using the Risk Ratio for binary outcomes. The second analysis was a calculation of an overall mean of TSH levels presented in each study as continuous variables, using the log transformed means and backtransformed to the original measurement scale.

In both meta-analyses a random effects model was the first choice considering the anticipated heterogeneity, and also a common effect model was used in order to provide a standpoint for effect comparison. The meta-analysis of proportions was performed using the Mantel–Haenszel method, the DerSimonian–Laird estimator to calculate the heterogeneity variance (τ^2^) with 95% confidence intervals (95% CI). The meta-analysis of single means was performed using the inverse variance method, the Hunter-Schmidt method to estimate the between-study variance (τ^2^) and its square root (τ), presented with 95% confidence intervals (95% CI). In both cases, the Knapp-Hartung adjustments were used to calculate the confidence interval for the summary effect due to the expected variance for the limited number of studies. In both cases, heterogeneity was estimated by the I^2^ (15), considering values of 50–75% as moderate heterogeneity, and values greater than 75% as high heterogeneity. The funnel and Baujat plots were used to check for sources of heterogeneity and the Egger test for asimmetry (16, 17).

A meta-regression was performed considering the gestational outcome variables, mean TSH, and risk of bias. The categorical moderators were predefined covering the TSH groups versus (a) clinical pregnancy, (b) mean TSH, (c) mean FSH, and (d) live birth rate. The results were presented as regression coefficients with 95% confidence intervals (CI), association *z* values, and *p* values.

The data were analysed using R Studio software, version 1.1.383 (The R Foundation for Statistical Computing, Vienna, Austria), using the *meta* (18) and the *metafor* packages (19).

#### Risk of bias assessment

The risk of bias assessment was performed by two independent investigators, with disagreements resolved by a third part (CPB). We used the ROBINS-E tool for risk of bias assessment, and the results are presented as charts according to the defined by the authors (20). Certainty is reported as 95% CI without previous definition of the limits considering that the protocols for IVF/ICSI are fully contextualized and performed under well-established settings of clinical practice guidelines (21).

## Results

### Retrospective study

A total of 1,016 women undergoing IVF/ICSI were evaluated for inclusion in this study. Of these, 256 women had the required data and met the inclusion and exclusion criteria. The thyroid function of euthyroid patients was evaluated by categorising the patients into two groups according to serum TSH levels: low-TSH group (<2.50 mIU/L) and high-TSH group (≥2.5 mIU/L). The clinical and hormonal parameters and gestational outcomes according to the TSH levels are presented in **Table 1**. No statistically significant differences were found between the groups for any analysed variable, including FT4 levels (p=0.473).

**Table 1:** Clinical characteristics, hormonal profile and reproductive outcomes of infertile women according to TSH levels.

Variables*	TSH levels				p-value
	≥0.5 ≤ 2.49 mUI/mL		≥2.5 ≤ 4.5 mUI/mL		
N	211	82.4	45	17.6	–
Age (years)	34	[32-37]	34	[32-37]	0.431^a^
BMI (Kg/m2)	22.7	[21.2-25]	22.6	[21.3-24.6]	0.489 ^a^
FSH (mUI/mL)	6.5	[5.3-7.4]	6.4	[5.5-7.4]	0.439 ^a^
LH (mUI/mL)	5.6	[4.1-6.9]	5	[3.9-7.7]	0.429 ^a^
FT4 (ng/dL)	1.1	[1-1.2]	1.1	[1-1.2]	0.473 ^a^
25(OH)D (ng/mL)	30	[23-40.2]	32.2	[25.4-42.1]	0.145 ^a^
Prolactin (ng/mL)	12.5	[8.6-17.4]	12.5	[9.4-16.9]	0.421^a^
AFC	13	[9-19]	14	[9-19]	0.322 ^a^
Total FSHr (UI)	2025	[1575-2400]	1800	[1575-2100]	0.369 ^a^
Oocytes retrieved	10	[6-15]	9	[6-13]	0.177 ^a^
MII	8	[5-12]	7	[5-11]	0.157 ^a^
Embryos	4	[3-6]	4	[3-6]	0.319 ^a^
Clinical Pregancy rate (n, %)	97	45.9	20	44.4	0.982^b^
Live-births (n)	121	57.3	23	51.1	0.225^b^
Twins (n, %)	27	27.8	3	15.0	0.365^b^
Pre-term (n, %)	13	13.4	3	15.0	0.99^b^

*Qualitative variables were presented by absolute and relative frequency, and quantitative variables by median and interquartil interval. 25(OH)D; 25-Hydroxyvitamin D, AFC; Antral Follicle Count, BMI; Body Mass Index: FSH; Follicle-stimulating hormone, FSHr; Recombinant FSH, FT4; free Thyroxine, LH; Luteinizing hormone, MII; Metaphase II oocytes, TSH; Thyroid stimulating hormone. aMann-Whitney U test, bChi-Square test.

To analyse the profile of women with favourable and adverse gestational outcomes, the patients were dichotomised according to the primary reproductive outcome into the groups: Clinical Pregnancy Yes and No. The data are shown in **Table 2**. Women who presented clinical pregnancy showed statically greater antral follicle count (AFC) (p<0.001), oocytes retrieved (p=0.032), metaphase II (MII) (p=0.012) and total embryos (p=0.004), besides being a little younger than women who did not achieve clinical pregnancy. However, no statistical difference regarding FT4 levels was found between the groups according to clinical pregnancy outcomes (p=0.376).

**Table 2:** Clinical characteristics, hormonal profile and reproductive outcomes of infertile women according to clinical pregnancy outcome.

Variables*	Clinical Pregnancy				p-value
	No		Yes		
N	139	–	117	–	–
Age (years)	35	[32-38]	33	[32-36]	**0.013^a^**
BMI (Kg/m2)	22.8	[21.4-25.3]	22.49	[21.1-24.5]	0.116^a^
FSH (mUI/mL)	6.4	[5.3-7.7]	6.49	[5.3-7.4]	0.429^a^
LH (mUI/mL)	5.2	[3.8-7]	6.07	[4.5-7]	0.055^a^
TSH (mUI/mL)	1.7	[1.2-2.2]	1.67	[1.2-2.3]	0.270^a^
FT4 (ng/mL)	1.1	[1-1.3]	1.13	[1.0-1.2]	0.376 ^a^
25(OH)D (ng/mL)	31.8	[24.4-41.4]	28	[22-40]	0.077^a^
Prolactin (ng/mL)	12.5	[8.4-17.6]	12.5	[9.0-16.6]	0.411^a^
AFC	12	[9-17]	15	[11-21]	**<0.001^a^**
Total FSHr (UI)	2025	[1575-2400]	1800	[1575-2100]	0.285^a^
Oocytes retrieved	9	[5-13.5]	11	[6-15]	**0.032^a^**
MII	7	[4-11]	9	[5-13]	**0.012^a^**
Embryos	4	[2-6]	5	[3-7]	**0.004^a^**

*Qualitative variables were presented by absolute and relative frequency, and quantitative variables by median and interquartil interval. 25(OH)D; 25-Hydroxyvitamin D, AFC; Antral Follicle Count, BMI; Body Mass Index, FSH; Follicle-stimulating hormone, FSHr; Recombinant FSH, FT4; free Thyroxine, LH; Luteinizing hormone, MII; Metaphase II oocytes, TSH; Thyroid stimulating hormone. ^a^Mann-Whitney U test. Bold means statistically significant p-value.

So, we stratified the women according to the age into two groups: women aged ≤35 years (n=154) and women aged >35 years (n=102). As expected, women aged ≤35 years presented significantly lower FT4 (p=0.002), and greater AFC (p=<0.001), oocytes retrieved (p=0.029), MII (p=0.015), embryos (p=0.002); clinical pregnancy rate (p=0.019), and live births (0.041) (data not shown).

**Figure 1:**
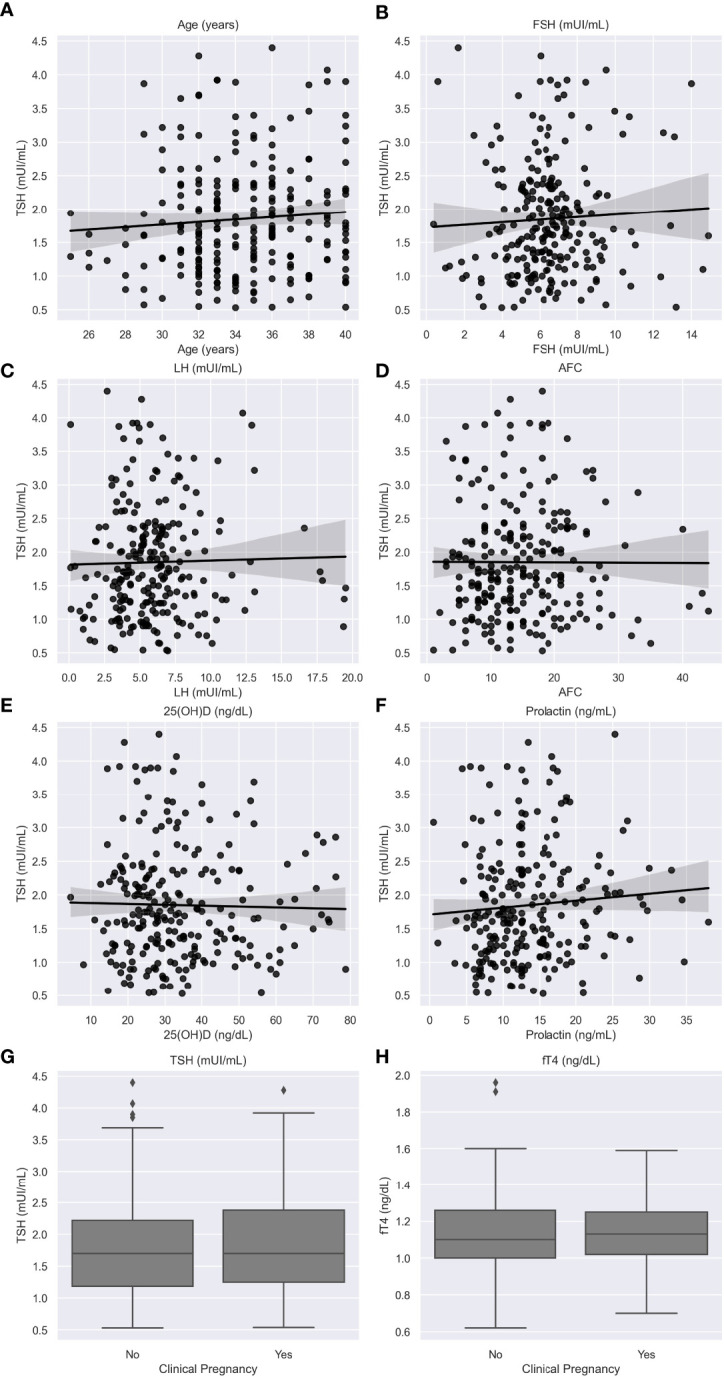
Relationship between TSH levels and age **(A)**; FSH levels **(B)**; LH levels **(C)**; 25(OH)D **(D)** levels; antral follicle count **(E)**; prolactin levels **(F)**; and clinical pregnancy **(G)**; and relationship between FT4 levels and clinical pregnancy **(H)**. Caption: 25(OH)D: 25-Hydroxyvitamin D; AFC: Antral Follicle Count; Follicle-stimulating hormone; FT4: free thyroxine; LH: Luteinizing hormone; TSH: Thyroid stimulating hormone. Black lines mean count and gray area the confidence interval.

The Spearman’s correlation test for serum TSH and FT4 levels of euthyroid women and variables of interest associated to clinical pregnancy showed no statistically significant correlations (**Table 3**), except for a weak inverse correlation between FT4 and BMI (p=0.049). Graphical representations of the associations are shown in **Figure 1**.

**Table 3:** Correlation of TSH and FT4, and factors associated to clinical pregnancy in euthyroid women undergoing IVF.

	Variables	Rho	p-value
TSH	Age	0.074	0.249
	BMI	0.07	0.278
	FSH	0.061	0.341
	FT4	-0.002	0.975
	LH	0.051	0.428
	25(OH)D	-0.029	0.647
	AFC	0.042	0.509
	Oocytes	0.009	0.886
	MII	0.013	0.842
	Embryos	0.072	0.259
	Clinical Pregnancy	0.035	0.588
FT4	Age	0.098	0.125
	BMI	-0.126	0.049
	FSH	-0.061	0.342
	TSH	-0.002	0.975
	LH	0.072	0.263
	25(OH)D	0.03	0.635
	AFC	-0.015	0.812
	Oocytes	-0.052	0.421
	MII	-0.025	0.702
	Embryos	-0.053	0.41
	Clinical Pregnancy	0.023	0.723

25(OH)D: 25-Hydroxyvitamin D; AFC: Antral Follicle Count; FSH: Follicle-stimulating hormone; FT4: free thyroxine; Spearman Correlation test.

### Systematic review and summary of evidence

**Figure 2:**
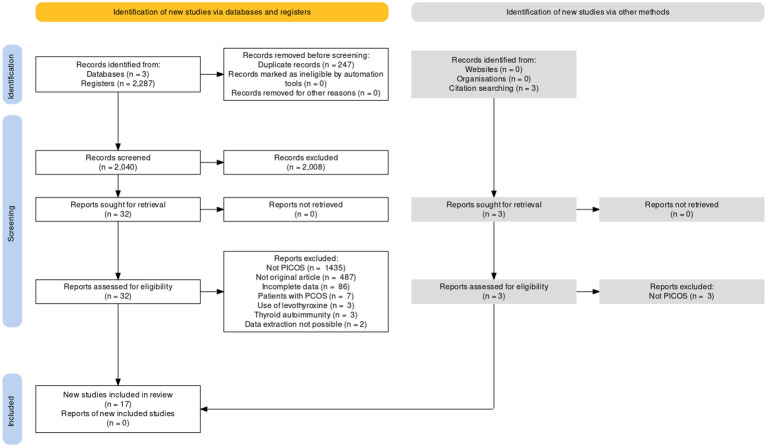
PRISMA flow for study selection.

A total of 17 articles were included in the meta-analysis, comprising 13.247 women undergoing IVF/ICSI (22-37, and the retrospective present study). **Figure 2** outlines study selection process in the PRISMA flow diagram.

#### Study characteristics

**Table 4** shows the characteristics of the studies included. All studies included were retrospective cohort studies. Most of the studies (10 of 17) had been conducted in non-Asian populations.

**Table 4:** Summary of evidence of the studies included.

Authors	Year	Country	Study Design	N	TSH groups	Pregnancy(N)	Delivery(N)	Main finding
Baker et al. (22)	2006	USA	Retrospective Cohort	364	≤2.5mIU/L=72 >2.5mIU/L=92	239 76	150 45	No difference in clinical pregnancy and live delivery
Reh et al. (23)	2010	USA	Retrospective Cohort	1055	≥2.5 mIU/L=248 <2.5 mIU/L=807	128 377	97 370	No difference in clinical pregnancy and live delivery
Michalakis et al. (24)	2011	USA	Retrospective Cohort	1500	0.4-2.5 mIU/L=842 2.5–4.0 mIU/L=278	368 123	295 97	No difference in clinical pregnancy and live birth
Ding et al. (25)	2012	China	Retrospective Cohort	372	<2.0 mIU/L=258 2.0-4.5 mIU/L=114	78 49	–	There was a significant difference in clinical pregnancy rate among the groups.
Aghahosseini et al. (26)	2014	Iran	Retrospective Cohort	816	<2.5mIU/L=487 ≥2.5mIU/L=329	131 78	–	The risk ratio of negative clinical pregnancy higher in TSH ≥ 2.5 mIU/L
Mintziori et al. (27)	2014	Greece	Retrospective cohort	158	≤2.5mIU/L=120 >2.5mIU/L=38	46 14	41 14	No difference in clinical pregnancy and live birth rates
Karmon et al. (28)	2015	USA	Retrospective cohort	1477	0.4-2.49mIU/L=1079 ≥2.5mIU/L=398	357 126	269 111	No difference in clinical pregnancy or live births rates
Weghofer et al. (29)	2015	USA	Retrospective Cohort	431	≤2.5μIU/mL=330 >2.5μIU/mL=101	73 15	46 5	A trend towards improved pregnancy potential in the presence of TSH ≤ 2.5 mIU/L
Coelho-Neto et al. (30)	2016	Brazil	Retrospective cohort	617	<2.5mIU/L=455 2.5- 4.0mIU/L=162	111 42	92 36	Similar reproductive outcomes, including live birth and clinical pregnancy rates
Gingold et al. (31)	2016	USA	Retrospective cohort	1090	0.5-2.5mIU/L=773 2.5-5mIU/L=317	461 224	256 118	No difference in the early pregnancy loss rate across in euthyroid patients
Tuncay et al. (32)	2018	Turkey	Retrospective Cohort	302	0.38–2.49 mIU/L= 233 2.50–4.99mIU/L= 69	26 12	24 12	TSH 2.5 and 4.9 mIU/L do not have a negative effect on clinical pregnancy nor intrauterine fetal death
Grove-Laugesen et al. (33)	2019	Denmark	Retrospective cohort	596	<2.5mIU/L=503 >2.5mIU/L=93	22 156	20 146	TSH level >2.5 mIU/L was associated with lower odds for clinical pregnancy and live birth
Turgay et al. (34)	2019	Turkey	Retrospective Cohort	156	0.5-2.49 mIU/L=118 2.5-4.5 mIU/L=38	18 4	14 4	No difference live birth, clinical pregnancy and miscarriage rates
Jin et al. (35)	2019	China	Retrospective cohort	1185	≤2.5mIU/L=830 >2.5mIU/L=355	441 175	–	No significant effect on the clinical pregnancy rate
Zhang et al. (36)	2020	China	Retrospective cohort	1786	0.27–2.5 mIU/L=1008 2.5–4.2 mIU/L=778	568 452	471 385	No difference in clinical pregnancy and live-birth
Karakis et al. (37)	2021	Turkey	Retrospective Cohort	1465	0.27–2.5 mIU/L= 1110 2.51–4.5 mIU/L= 355	96 28	90 25	TSH between 2.5 and 4.5 mIU/L is not associated with lower pregnancy rates or live birth rates
Present study	2022	Brazil	Retrospective cohort	256	0.5-2.49 mIU/L=211 2.5-4.5 mIU/L=45	97 20	94 20	No difference in clinical pregnancy or live-birth rates

#### Risk of bias in the studies

**Figure 3:**
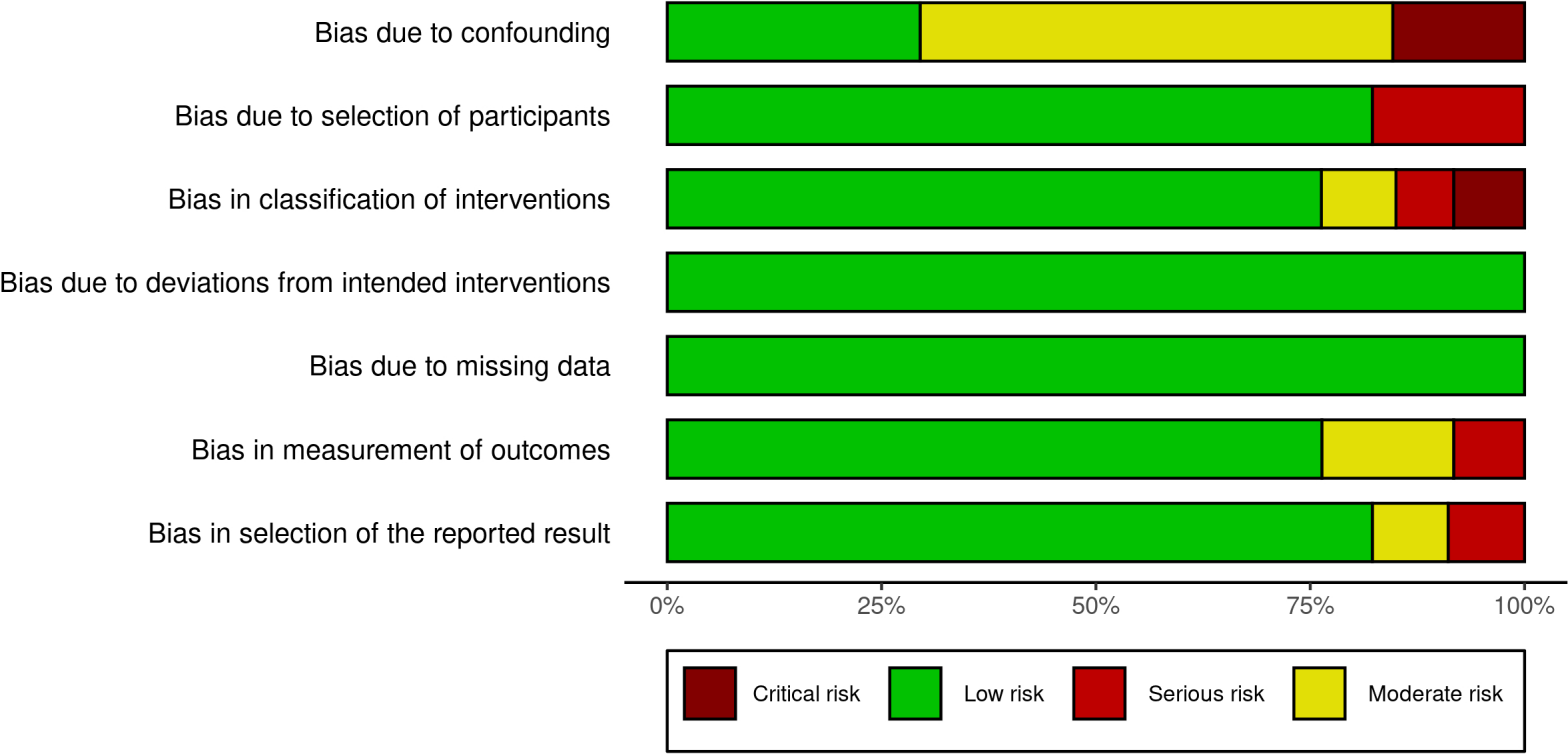
ROBINS-I Summary plot.

**Figure 4:**
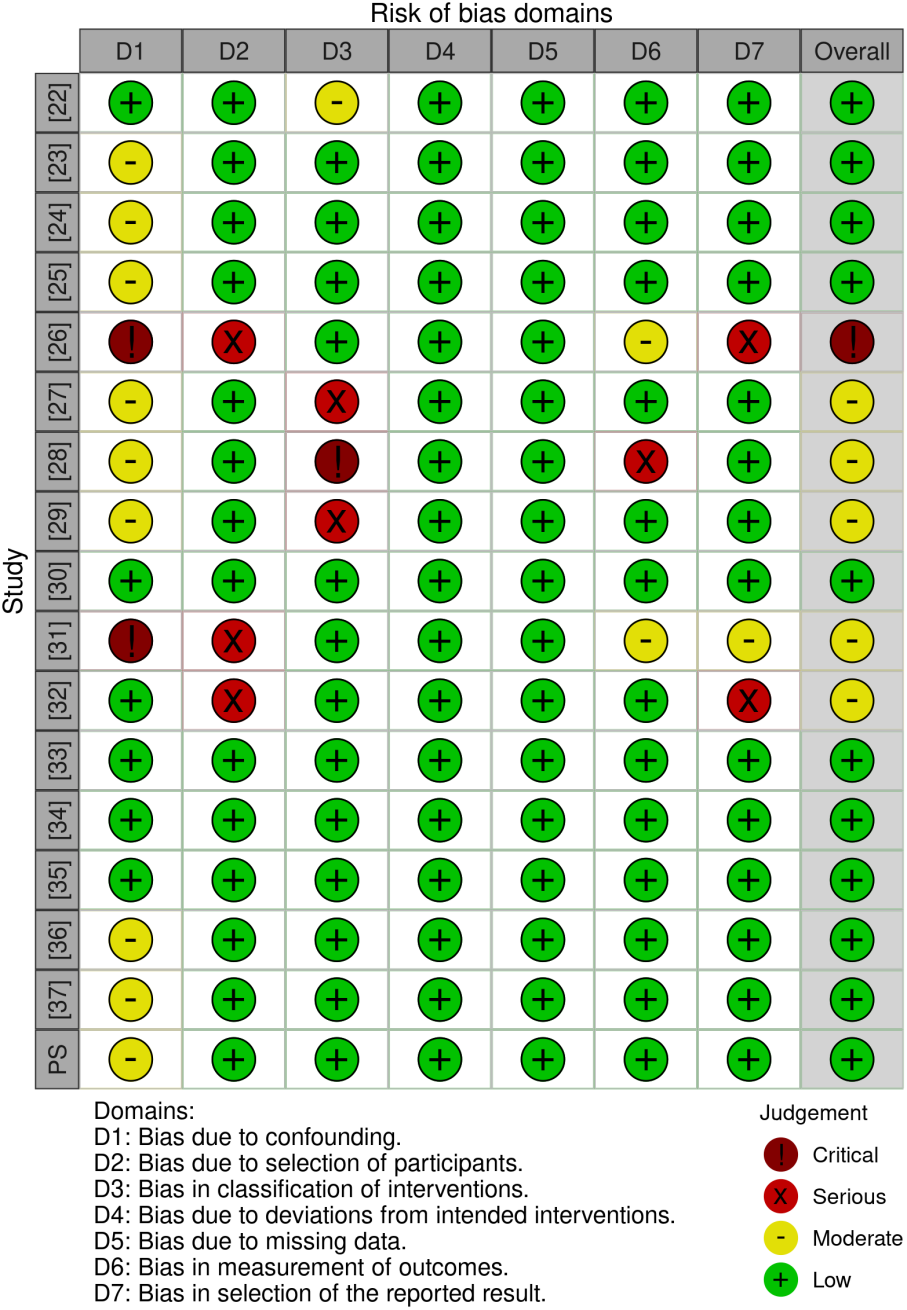
ROBINS-I for Individual studies. Caption: Within parentheses, the number represents the study reference. PS: Present study.

According to the ROBINS-E tools, only one study presented critical risk of bias (26). The domain 1 (risk due to confounding) is expected due to intrinsic factors regarding the nature of the FIV/ICSI procedure. Other than this, domains regarding the outcomes measurement present a low risk. Moreover, considering that the studies included are observational, the overall risk of bias ranging from low to moderate is a positive finding (**Figures 3** and **4**).

#### Results of syntheses

**Figure 5:**
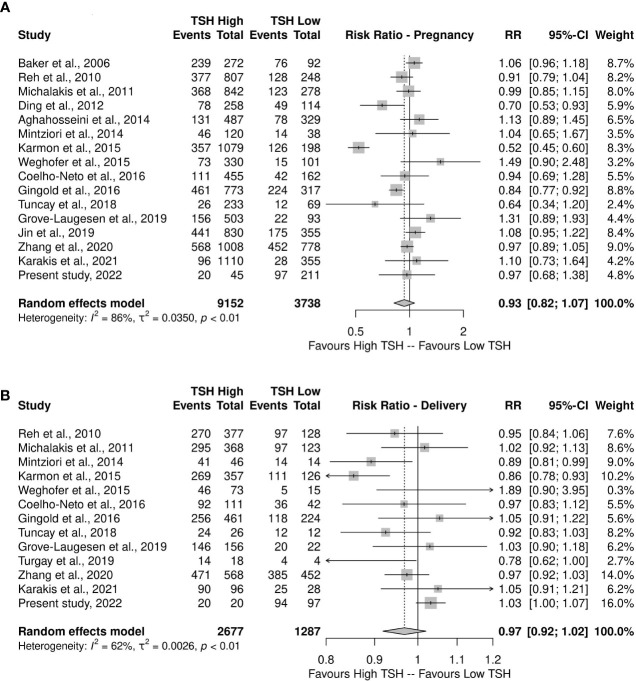
**(A)** Meta-analysis of proportions of pregnancies (Events) from the total number of patients comparing groups with low versus high TSH **(B)** and delivery (Events) from the total number of pregnancies (Total) comparing groups with low versus high TSH.

An analysis of proportions between the 0.5–2.49 mIU/L and 2.5–4.5 mIU/L TSH groups in relation to the primary outcome of clinical pregnancy revealed a very small difference between the two groups’ gestational outcomes (RR 0.93, 95% CI 0.80–1.08), although there was a greater tendency for pregnancy in patients with TSH between 2.5 and 4.5 mIU/L (**Figure 5A**). High heterogeneity was observed among studies (I^2^: 78%; τ^2^: 0.0544; p<0.01).

The comparison between the TSH groups regarding deliveries showed a higher occurrence of this outcome in the high-TSH group (RR 0.97, 95% CI 0.92–1.02), with moderate heterogeneity (I^2^: 62%; τ^2^: 0.0026; p<0.01), as observed in **Supplementary Figure 1** and confirmed by the funnel-plot asymmetry test (t=-1.01, df=9, p=0.3388). However, the lower heterogeneity may be attributed to larger confidence intervals of estimates. Nevertheless, the result is similar to the pregnancy rate, both with risk ratio values and CI suggesting no actual difference between the groups (**Figure 5B**).

#### Subgroup analysis

A sensitivity analysis was performed comparing the mean TSH concentrations in the two groups, low dispersion was found between the studies, both individually and comparatively (I^2^: 100%; τ^2^: 0.1011) (**Supplementary Figure 2**).

#### Publication bias

The funnel and Baujat plots indicate a high degree of publication bias (**Supplementary Figure 3**), with an asymmetry confirmed by the Egger test (t=0.40, df=12, p=0.6971).

## Discussion

The present retrospective study found no statistically significant difference when evaluating TSH and FT4 as a predictor of gestational outcome. Other factors of interest investigated, such as AFC, retrieved oocytes, and MII, which showed significant differences between the group that achieved clinical pregnancy and the group that did not, have already been explored in the current literature and are well established as predictors of gestational outcome in IVF/ICSI (38–40). The findings of the categorisation according to age also agree with the existing literature by confirming the maternal age as an important predictor of ovarian reserve (38).

As for the systematic review and meta-analysis, no significant difference was observed in clinical pregnancy rates between groups of euthyroid women whose serum TSH levels were <2.5 mIU/L and ≥ 2.5 mIU/L. Also, no significant difference was found when comparing the mean live births in the two groups, although the frequency of deliveris was higher in euthyroid women with TSH levels of ≥2.5 mIU/L.

Some guidelines and review studies suggest that it is preferable for a woman who wishes to become pregnant to maintain serum TSH concentrations <2.5 mIU/L, and this level should be preserved during the first and second trimesters of pregnancy (41, 42). The harm of TSH levels above normality (>4.5 mIU/L) is attested by some studies that, by means of multivariate analysis, found a positive correlation between high TSH levels and decreased clinical pregnancy rates, although the same correlation has not been attested for the frequency of miscarriages and live births (25, 43). Hyperthyroidism, marked by low serum TSH levels, is also related to decreased rates of live births (5). Nevertheless, evidence suggests that TSH variations within the normal range do not *per se* cause differences in the gestational outcomes (27, 44). In one of the studies used in this review, TSH levels higher than 2.5 mIU/L but still within the normal range were found to be positively associated with the number of live births (28). This result was also found in the proposed meta-analysis, although no statistical significance was found.

In studies that used other stratifications of normal TSH values, there were also no differences in the clinical pregnancy or live birth rates (30, 45). A study using groups whose patients presented pre-COS TSH levels <2.5 mIU/L and were later stratified into those with levels below and above that value investigated the gestational outcomes and did not find any statistical difference between the groups (46). Therefore, the role of TSH in reproductive outcomes appears to be unimportant when within normal levels in patients without autoimmune diseases, as suggested by this retrospective study and review.

A tendency for a higher percentage rate of clinical pregnancy can be noticed in groups of women with low TSH, albeit without statistical support (29, 34, 47), although there are studies supporting similar percentage values or even favouring the high TSH group (23, 32, 48). Other authors support the thesis that LT4 supplementation in euthyroid women with TSH >2.5 mIU/L should be initiated only after clinical pregnancy and not during the pre-conception period (49). Apparently, the use of LT4 alters reproductive outcomes in women with normal TSH, as one study found a significant correlation of pre-conception TSH levels between 2.5 and 4.5 mIU/L with adverse gestational outcomes in the group of women taking LT4 supplementation, whereas the same correlation was not observed in the arm of the study in which patients who did not use the drug were excluded (33). However, when analysing patients who did not use LT4, that study found an apparently higher pregnancy rate in the high-TSH group, albeit without statistical significance.

Another review and meta-analysis on this subject suggested that clinical pregnancy rates were lower in euthyroid women with TSH above 2.5 mIU/L, although the results were not statistically significant (35). In the present study, this trend was not observed, but rather its opposite was observed, not only for clinical pregnancy rates, but also for the frequency of live births.

The present study chose to exclude patients with autoimmune thyroiditis, because evidence shows that levels of thyroid autoantibodies in the follicular fluid are not associated with changes in gestational outcomes (9, 27, 48, 50, 51), although there are studies proposing the opposite (47, 52). However, there is a lack of studies evaluating gestational outcomes from positive and negative groups for anti-thyreoperoxidase in relation to subgroups of normal TSH variations.

The interference of TSH levels on the ovarian reserve, retrieved oocytes, and AFC was not measured in the present meta-analysis, even though there are reports that reinforce that high TSH levels impact these variables (24). Few studies analysed in the present review had this information available.

The main limitation in our retrospective study was the number of patients lost during the selection stages. Moreover, the authors cannot guarantee that the patients do not have any autoimmune disease, but those who have were excluded, and all patients have no autoimmune thyroiditis. In addition, there was a discrepancy between the sample size of the low- and high-TSH groups. However, the problem of unbalanced groups is critical for small samples with normal distribution, where tests are more sensitive to biases. In the present study, the non-normal distribution of data indicated the use of non-parametrical tests which are less sensitive to such differences due to the expected distortions in the data distribution. Moreover, considering that it is an observational retrospective study, we aimed to adopt the most restrictive selection criteria, and inevitably, it lowered the sample size. Nevertheless, the power analysis resulted in a power of 84.3%. The limitations found in the systematic review and meta-analysis was especially due to the heterogeneity of the selected publications, whether in sample size, methodology used, or presentation of the results. Although the scores indicated by the ROBINS-E tool did not present important variations and were not particularly low, it was possible to identify limitations in the studies, such as the considerable sample imbalance between TSH levels groups in the analyses performed.

It should be noted that despite international recommendations to maintain TSH levels <2.5 mIU/L in women with hypothyroidism, the present study addressed euthyroid women without autoimmune thyroid disease submitted to assisted reproduction techniques. Therefore, for this group of patients, the results presented do not support encouraging treatment with FT4 with the aim to lower the TSH range between 2.5 and 4.0 mIU/L to a target <2.5 mIU/L.

In summary, TSH levels <2.5 mIU/L in euthyroid women without autoimmune disease undergoing IVF/ICSI do not appear to result in higher rates of clinical pregnancy or deliveries. If anything, in our meta-analysis there were higher rates of pregnancy and delivery in patients with normal TSH values >2.5 mIU/L, but this trend did not achieve statistical significance. To confirm the results, observational studies with a larger number of patients and methodological standardisation are recommended to enable better investigation and comparison.

## Data availability statement

The raw data supporting the conclusions of this article will be made available by the authors, without undue reservation.

## Ethics statement

The design, analysis, interpretation of data, drafting, and revisions followed the Helsinki Declaration, the Preferred Reporting Items for Systematic Reviews and Meta-Analyses (PRISMA) and the Meta-analysis of Observational Studies in Epidemiology (MOOSE) guidelines. This study is registered in International Prospective Register of Systematic Reviews (PROSPERO) under the CRD 42022306967 and it was approved by the Research Ethics Committee of the Centre for Medical Sciences of the Federal University of Paraíba (approval code CAAE 25654719.5.0000.8069). Signing the Informed Consent Form was waived by the Research Ethics Committee.

## Author contributions

Conceptualization: VD’A and BB. Methodology: VD’a, EM, RO and BB. Software: EM. Validation: EM, RO and BB. Formal analysis: VD’A, EM and BB. Investigation: VD’A, EM, LD’A, MC and RN. Resources: MC, EM and BB. Data curation: VD’A, EM and RN. Writing – original draft preparation: VD’A, LD’A and RN. Writing – review and editing: BB, RO, DC, AL and CB. Supervision: BB and RO. Visualization: VD’A, EM and BB. Project administration: BB. All authors critically reviewed the manuscript and approved the final version of the manuscript. All authors contributed to the article and approved the submitted version.

## Acknowledgments

We would like to thank all the individuals who participated in this study and supported this research.

## Conflict of interest

The authors declare that the research was conducted in the absence of any commercial or financial relationships that could be construed as a potential conflict of interest.

## Publisher’s note

All claims expressed in this article are solely those of the authors and do not necessarily represent those of their affiliated organizations, or those of the publisher, the editors and the reviewers. Any product that may be evaluated in this article, or claim that may be made by its manufacturer, is not guaranteed or endorsed by the publisher.

## Supplementary material

Supplementary Figure 1
**(A)** Funnel plot - proportions for low x high TSH by pregnancy, and **(B)** Baujat plot - proportions for low x high TSH by pregnancy.

Supplementary Figure 2 Forest plot - Meta-analysis of means: mean TSH for pregnancy (subgroup low and high TSH).

Supplementary Figure 3
**(A)** Funnel plot - proportions for low x high TSH by delivery rate, and **(B)** Baujat plot – proportions for low x high TSH by delivery rate.

## Abbreviations

25(OH)D, 25-hydroxyvitamin D; AFC, Antral Follicle Count; CI, Confidence Intervals; COS, Controlled Ovarian Stimulation; FSH, Follicle-Stimulating Hormone; FT4, Free Thyroxine; GnRH, Gonadotropin Release Hormone; IQR, Interquartile Ranges; IVF/ICSI, in Vitro Fertilisation/Intracytoplasmic Sperm Injection; LH, Luteinising Hormone; LT4, Levothyroxine; MII, Meiosis II Stage; OR, Odds Ratios; PRISMA, Preferred Reporting Items for Systematic Reviews and Meta-Analyses; RR, Relative Risk; TSH, Thyroid-stimulating hormone.
