# Supplementary figures and images for: Effect of thyroid function on assisted reproduction outcomes in euthyroid infertile women: A single center retrospective data analysis and a systematic review and meta-analysis
Source: Front Endocrinol (Lausanne). 2022 Oct 10;13:1023635. doi: 10.3389/fendo.2022.1023635 (PMC9589421; doi:10.3389/fendo.2022.1023635)

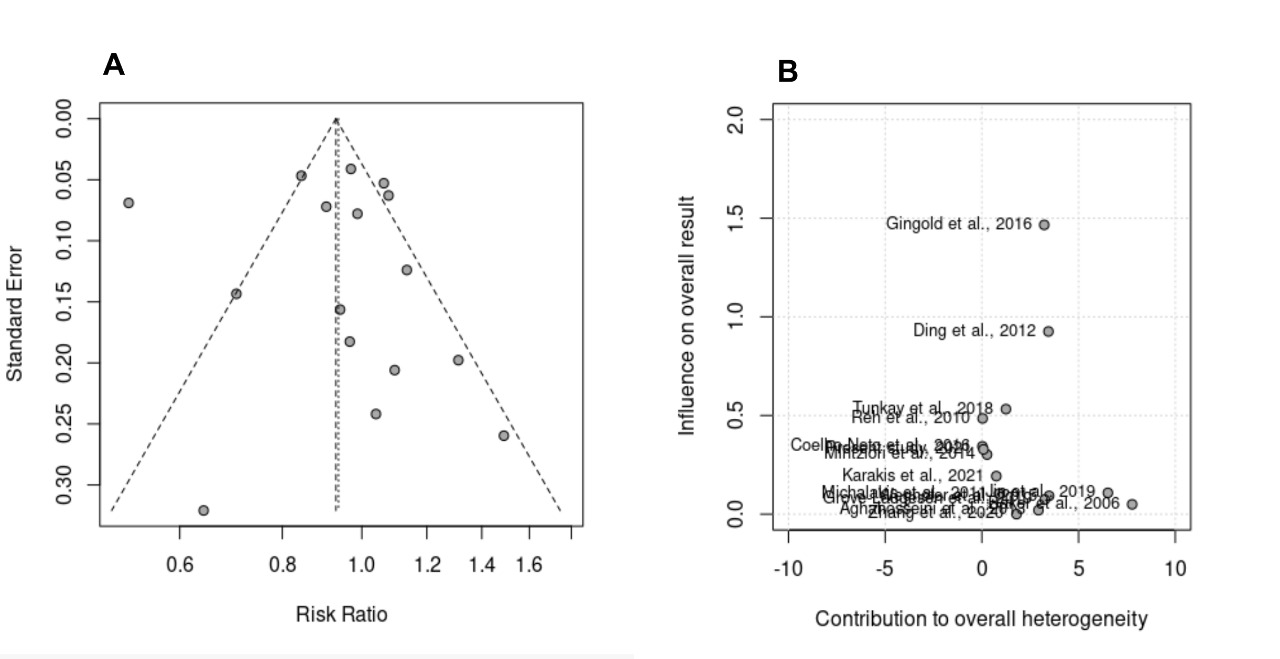

Supplement: Supplementary Figure 1 — (A) Funnel plot - proportions for low x high TSH by pregnancy, and (B) Baujat plot - proportions for low x high TSH by pregnancy. [file Image_1.tiff]

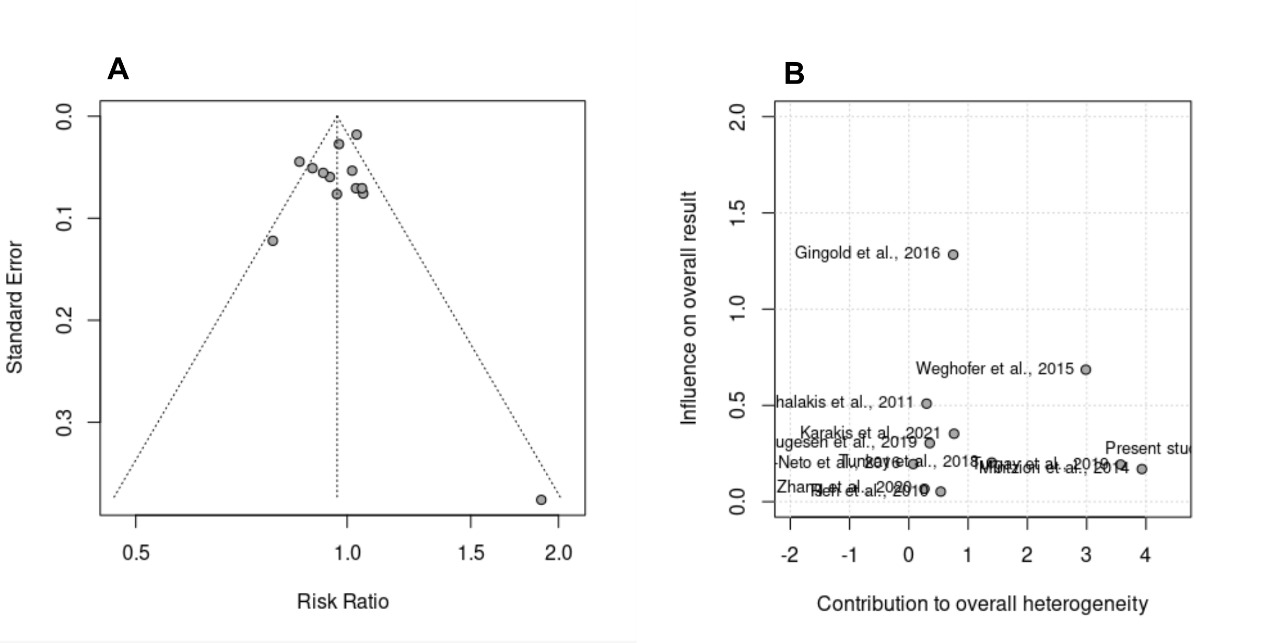

Supplement: Supplementary Figure 3 — (A) Funnel plot - proportions for low x high TSH by delivery rate, and (B) Baujat plot – proportions for low x high TSH by delivery rate. [file Image_3.tiff]
